# A novel surgical technique for cranial cruciate ligament repair in dogs using autologous lateral digital extensor muscle tendon graft combined with platelet-rich plasma: A preliminary experimental study

**DOI:** 10.14202/vetworld.2025.210-219

**Published:** 2025-01-30

**Authors:** Mousa H. Daradka, Mays A. Malkawi, Zuhair Banni Ismail, Hanan M. Hammouri, Mahmoud A. Abu-Abeeleh, Saba A. Rihani

**Affiliations:** 1 Department of Clinical Veterinary Medical Sciences, Faculty of Veterinary Medicine, Jordan University of Science and Technology, P.O. Box 3030, Irbid, 22110, Jordan; 2 Department of Mathematics and Statistics, Faculty of Science and Arts, Jordan University of Science and Technology. P.O. Box 3030, Irbid, 22110, Jordan; 3 Deparment of General Surgery, Faculty of Medicine, The University of Jordan.P.O.Box 13857, Amman, 11942, Jordan

**Keywords:** canine, cranial cruciate ligament, PRP, tendon graft, tissue healing

## Abstract

**Background and Aim:**

Cranial cruciate ligament (CrCL) injuries are a prevalent orthopedic issue in dogs, typically managed through surgical interventions such as tibial plateau leveling osteotomy and tibial tuberosity advancement. However, these techniques have limitations, including high costs and extended recovery periods. This study introduces an innovative CrCL repair method employing an autologous lateral digital extensor muscle tendon graft and evaluates the effects of platelet-rich plasma (PRP) on tissue healing.

**Materials and Methods:**

Twenty-four healthy, male, local-breed dogs were divided into two groups. Group A underwent the surgical procedure without PRP, while Group B received intra-articular PRP during surgery. Outcomes were evaluated through clinical assessments of lameness, post-operative complications, and histological analysis over 10, 20, 30, and 40 days.

**Results:**

The PRP-treated group demonstrated statistically significant improvements in post-operative complication scores (p = 0.0025) and histological outcomes (p = 0.0002). However, graft maturation was unaffected by PRP treatment but improved over time (p = 0.0013). PRP-treated dogs exhibited faster recovery and enhanced tissue regeneration, with reduced inflammation and improved graft-bone attachment.

**Conclusion:**

This novel surgical approach demonstrates significant potential for improving outcomes in CrCL repair by combining autologous tendon grafting with PRP. The technique offers reduced complications and enhanced healing, providing a promising alternative to traditional methods. Further studies are recommended to validate its efficacy in clinical settings.

## INTRODUCTION

Cranial cruciate ligament (CrCL) injuries in dogs are significant orthopedic conditions that can be influenced by both internal factors such as breed, age, sex, and genetics, as well as external biomechanical factors, such as joint conformation, bone alignment, and muscle strength [1, 2]. Modern canine lifestyles, particularly in Western societies, intensify these risks. Sedentary behavior weakens muscles and ligaments essential for joint stability, whereas intense sporadic activity places excessive strain on the musculoskeletal system, increasing the likelihood of CrCL injury [3, 4]. Nutritional factors and obesity also significantly contribute to CrCL rupture. Obese dogs face higher joint stress and inadequate nutrition during puppyhood, especially in large breeds, can lead to abnormal skeletal development, increasing the risk of CrCL injuries [2, 5]. Certain breeds, such as Labrador Retrievers and Rottweilers, are particularly vulnerable to CrCL disease, with Rottweilers often developing bilateral CrCL disease at a younger age [6]. Neutered dogs, particularly spayed females, are at higher risk of CrCL tears [6].

Conventionally, various surgical techniques are used for CrCL rupture repair, including lateral suturing, tibial plateau leveling osteotomy (TPLO), and tibial tuberosity advancement (TTA) [7]. These procedures require high surgical skill and availability of specialized implants, incur significant costs, and are associated with long recovery times. Platelet-rich plasma (PRP) has gained significant attention in veterinary orthopedics because of its potential to accelerate tissue healing and improve outcomes in musculoskeletal injuries, including CrCL ruptures [8]. PRP is rich in growth factors, cytokines, and other bioactive molecules that promote tissue regeneration, reduce inflammation, and stimulate cell proliferation [8]. The rationale for incorporating PRP in the treatment of CrCL injuries is twofold: First, it enhances the healing of the autologous tendon graft by promoting faster integration with the surrounding tissue and improving the tensile strength of the graft. Second, PRP’s anti-inflammatory properties may reduce post-operative swelling and pain, facilitating a faster recovery process, and reducing the risk of complications such as fibrosis and scar tissue formation. The dual purpose of PRP as both a healing enhancer and an adjunct to the surgical technique makes it a promising addition to the treatment regimen for CrCL ruptures, potentially improving both short- and long-term outcomes in affected dogs.

In dogs, the lateral digital extensor muscle tendon plays a critical role in stabilizing the hindlimb by facilitating the extension of the toes [9]. Despite its functional importance, the tendon is not directly involved in the major weight-bearing actions of the limb, such as walking, running, or jumping. As a result, its contribution to limb motion is minimal compared with that of other primary tendons. This characteristic makes it an ideal candidate for surgical repair, where its harvesting does not significantly affect the normal gait or functionality of the limb. Using this tendon in the surgical repair of CrCL rupture, it serves as an autograft, helping to restore joint stability without compromising the dog’s ability to perform basic movements.

Therefore, this study aimed to develop a novel surgical technique using an autologous lateral digital extensor muscle tendon graft and to evaluate the impact of PRP on the healing process in dogs with experimentally induced CrCL rupture.

## MATERIALS AND METHODS

### Ethical approval

The study protocol was reviewed and approved by the Institutional Animal Care and Use Committee of Jordan University of Science and Technology (Number 2019/342). Humane euthanasia procedures were also reviewed and approved by this committee.

### Study period and location

The study was conducted from July 2019 to January 2020 at the Veterinary Health Center at Jordan University of Science and Technology.

### Animals and study design

The study involved 24 intact local Mongrel male dogs with a mean age of 11.4 months and an average weight of 18 kg. The dogs were obtained from a local breeder. All dogs were clinically evaluated to confirm their health status and underwent necessary vaccinations and deworming before the study commenced. The animals were housed individually, and proper care and handling was provided by trained staff following ethical animal care guidelines.

Dogs were randomly divided into two groups of 12: Group A (control) underwent a novel surgical procedure for CrCL rupture repair and Group B (PRP-treated) received the same surgery along with intra-articular and tunnel injections of autologous PRP. Both groups were subdivided into subgroups based on the post-operative evaluation days (10, 20, 30, and 40 days).

### Follow up

In this study, randomization was conducted using a blind draw of numbered cards to assign dogs to groups and subgroups.

### Anesthesia

Pre-anesthetic evaluation, including blood testing, was conducted to ensure that each dog was healthy and fit for anesthesia and surgery. All dogs were fasted for 12 h before surgery, and access to water was allowed for 2 h before the procedure. General anesthesia was induced using 2% xylazine (1.1 mg/kg body weight) followed by 10% ketamine hydrochloride (15 mg/kg body weight) intramuscularly [11]. Once anesthetized, left lateral stifle radiographs were obtained to confirm the joint normalcy.

### Surgical procedure

After anesthesia, the dogs were positioned in the right lateral recumbency on the surgical table. The left hind limb was shaved, scrubbed using povidone-iodine solution and sterile saline, and draped for aseptic surgery [9].

### Harvesting lateral digital extensor muscle tendon

Three skin incisions were made to harvest the lateral digital extensor muscle tendon [9, 10]. The first was a 2-cm incision made laterally at the level of the lateral tibial malleolus on the lower third of the tibia. The tendon was identified, exteriorized, and severed at the lowest possible point near the hock joint. The second incision (3 cm long) was made laterally at the mid-third of the tibia over the interosseous space. A final 10-cm curved incision was made craniolaterally, centered over the patella, and extended through the subcutaneous tissue to expose the septum between the fascia lata and the biceps femoris muscle proximally and the lateral retinaculum distally. The tendon was pulled dorsally through the first and second incisions using straight artery forceps. After being pulled through the third incision, the first and second incisions were closed routinely using a 2-0 absorbable suture material. The pedicular graft was carefully dissected and cleaned of excess tissue and fat and placed in sterile saline-soaked gauze until implanting for CrCL rupture repair.

### CrCL removal

A lateral parapatellar arthrotomy was performed to remove the CrCL [9-11]. A 10-cm craniolateral skin incision centered over the patella was made, followed by an incision through the subcutaneous tissue to expose the septum between the fascia lata and the biceps femoris muscle proximally and the lateral retinaculum distally. The same incision was used to exteriorize the tendon graft. The fascia lata was incised proximally and distally, continuing through the lateral retinaculum.

Once the joint capsule was visible, it was incised, and the incision extended proximally along the border of the vastus lateralis toward the fabella. The patella and fat pad were displaced medially to expose the cranial surface of the joint. After inspecting the joint, the CrCL was cut and removed to simulate complete CrCL rupture.

### CrCL rupture repair using a lateral digital extensor muscle tendon pedicular graft

**Figure 1:**
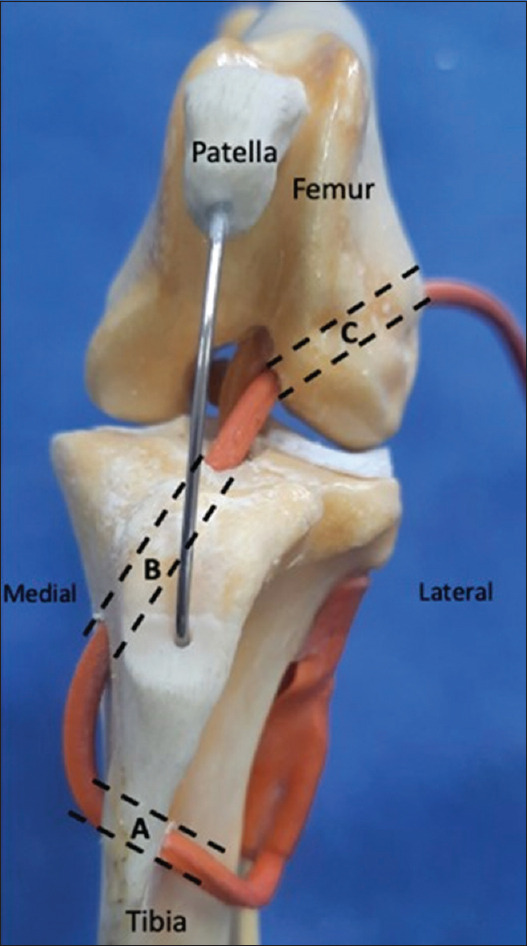
Illustration of the tunnels made for the lateral digital extensor tendon graft. (a) Tibial transverse tunnel made latero-medially within the tibial crest. (b) Tibial oblique tunnel made through the medial condyle of the tibia to the medial aspect of the cranial intercondyloid area of the tibia (which represents the insertion of the cranial cruciate ligament [CrCL]). (c) Represent the femoral oblique tunnel, which extends from the caudolateral aspect of the lateral femoral condyle to the craniomedial aspect of the intercondyloid fossa of the femur (which simulates and holds the forces of the CrCL).

The novel CrCL rupture repair procedure was performed through a third incision, which was also used to exteriorize the lateral digital extensor muscle tendon and perform the lateral parapatellar arthrotomy (Figure 1). After the CrCL was removed, a 3.5-mm transverse tunnel was drilled latero-medially within the tibial crest, 3 cm away from the stifle joint, using a 3.5-mm drill bit and a double-sided drilling guide. The tendon of the lateral digital extensor muscle was threaded into the tibial crest transverse tunnel using a K-wire guide pin and fully pulled from the lateral to the medial side of the tibial crest. Next, an oblique tunnel was drilled through the medial condyle of the tibia into the medial aspect of the cranial intercondyloid area of the tibia, representing the original insertion of the CrCL. This was performed using a 3.5-mm drill bit and a C-shaped drilling guide while the stifle joint was flexed. The tendon was threaded through this tibial oblique tunnel, passing from the medial tibial condyle to the cranial intercondyloid area within the stifle joint. Another oblique tunnel was drilled from the caudolateral aspect of the lateral femoral condyle to the craniomedial aspect of the intercondyloid fossa of the femur. This model was designed to simulate and bear the forces typically handled by the CrCL. The tunnel was drilled using a 3.5-mm drill bit and a C-shaped drilling guide. The tendon of the lateral digital extensor muscle was threaded through this femoral oblique tunnel, starting from the intercondyloid fossa caudomedially to the lateral femoral condyle caudolaterally, using a K-wire guide. The joint was then thoroughly irrigated with normal saline to remove bone debris, and the fat pad and patella were repositioned to their original locations.

After washing the joint, the end of the lateral digital extensor muscle tendon was debrided and pulled while the stifle joint was fully extended. The tendon was then anchored to the stifle joint capsule using 2-0 absorbable suture material with a simple interrupted suture pattern.

After securing the tendon, the joint capsule was closed with 2-0 absorbable sutures in a simple interrupted pattern. Joint stability was confirmed using the anterior drawer test. The subcutaneous and cutaneous layers were then closed routinely, with the subcutaneous layer sutured using a simple continuous pattern and the cutaneous layer sutured using a simple interrupted pattern, with 2-0 absorbable sutures. Finally, the incision site was covered with a non-adhesive bandage to protect the area.

### PRP preparation and administration

For dogs in Group B (PRP-treated), after general anesthesia was achieved, 20 mL of blood was drawn through jugular vein puncture using a 20 G 10 mL syringe and placed into EDTA tubes. The PRP was then prepared through differential centrifugation, following a previously described method by Dhurat and Sukesh [12]. The composition of the collected PRP was determined and compared with whole blood values [13]. The number of PRP was then adjusted to 1 million/mL [14].

In Group A, all dogs underwent lateral digital extensor muscle tendon harvesting, CrCL removal, and CrCL repair using a lateral digital extensor muscle tendon pedicular graft. No PRP was administered to dogs in this group. After repair of the CrCL rupture, 1 ml of sterile saline was administered intra-articularly into the oblique tibial and femoral tunnels immediately before the closure of the incision.

In Group B, all dogs underwent the same procedures as those in Group A, including lateral digital extensor muscle tendon harvesting, CrCL removal, and CrCL repair with the lateral digital extensor muscle tendon pedicular graft. After repair of the CrCL rupture, 1 mL of PRP (1 million/mL) was administered intra-articularly into the oblique tibial and femoral tunnels immediately before the closure of the surgical site.

### Post-operative care

Immediately after surgery, left lateral stifle radiographs were obtained to assess the stifle and bone tunnels. Then, each dog was monitored until it fully recovered. Once fully recovered, the dogs were transferred to their homes. Standard post-operative care included administering amoxicillin (10 mg/kg) intramuscularly twice daily for 7 days, as well as meloxicam (0.2 mg/kg) subcutaneously once on the day of surgery, followed by orally 0.1 mg/kg once daily for the next 4 days. In addition, tramadol was given at 3 mg/kg orally every 8–12 h for a week. All dogs were subjected to exercise restrictions throughout the experiment and kept on cage rest. The surgical area was protected with a non-adhesive bandage, and the incision was maintained daily for 2 weeks.

### Clinical evaluation

#### Lameness evaluation

Lameness evaluations were conducted on post-operative days 10, 20, 30, and 40. Lameness was classified using a previously published clinical scoring system by Goh [15], with grades ranging from 0 (no detectable lameness) to V (severe, predominantly non-weight-bearing lameness) (Table 1). The evaluation of lameness was performed by a blinded individual.

**Table 1:** Lameness scoring system used in this study.

Score	Characteristics
0	No detectable lameness at any gait
1	Barely perceptible lameness
2	Mild or inconsistently apparent weight-bearing lameness
3	Moderate weight-bearing lameness
4	Severe, predominantly weight-bearing lameness
5	Severe, predominantly non-weight-bearing lameness

### Post-operative complications

A scoring system was developed to assess the post-operative complications associated with this novel surgical procedure (Table 2). This system was used to evaluate dogs based on four parameters: Swelling, surgical site infection, pain, and the presence of crepitation during flexion or extension of the stifle joint. Each parameter was scored on a scale from 0 to 3, where 0 indicated no complication, 1 represented a mild degree of the complication, 2 denoted a moderate degree, and 3 signified a severe degree.

**Table 2:** Post-operative complication scoring system used in the study.

Score	Swelling	Infection	Pain	Crepitation
0	Absent	Absent	Absent	Absent
1	Mild	Mild	Mild	Mild
2	Moderate	Moderate	Moderate	Moderate
3	Severe	Severe	Severe	Severe

### Euthanasia

Dogs were humanely euthanized on days 10, 20, 30, and 40 postoperatively. For euthanasia, each dog was sedated with an intramuscular injection of 2% xylazine HCL at 1.1 mg/kg and 10% ketamine at 15 mg/kg and then euthanized through an intravenous injection of 80 mg/kg of 2.5% thiopental sodium. The 10-day euthanasia intervals were chosen to allow for a timely investigation of the healing process based on the typical healing timeline for CrCL rupture repair and the expected effects of PRP on tissue regeneration [16].

### Surgical site evaluation and sample collection

Following euthanasia, the left hind limbs were immediately dissected, photographed, and evaluated. The gross evaluation was performed on the joint, tunnels, and surgical site, examining graft integrity, synovial sealing at tunnel apertures, graft vitality, and the presence of the graft in the bone tunnels. These criteria were scored based on a previously published scoring system by Hexter *et al*. [17], with some modifications. Briefly, the graft thickness and apparent tension criteria were substituted with graft vitality. Synovial sealing at tunnel apertures criteria was substituted with graft in tunnels criteria to fit the surgical procedure that was done in the study. The classification of the modified post-mortem scoring system is detailed in Table 3. For graft integrity, a score of 0 indicates a complete rupture, defined as a complete loss of continuity of all fibers; a score of 1 denotes a partial rupture, with loss of continuity of some fibers; and a score of 2 represents no rupture, where there is no loss of fiber continuity. For synovial sealing at the tunnel apertures, a score of 2 is given when there is more than 75% circumferential sealing. A score of 1 is assigned for partial sealing, with 25%–75% of the circumferential sealing being assigned. A score of 0 is given for circumferential sealing of less than 25%. For graft vitality, a score 1 indicates a vital graft and 0 indicates a non-vital graft. For graft presence in the tunnels, a score of 3 was assigned for the presence of the graft in all three tunnels. Score 2 for the presence of the graft in two tunnels, a score of 1 was assigned for the presence of the graft in one tunnel, and a score of 0 was assigned if no graft was present in any tunnel. The overall graft maturation score was calculated from the separate scores, ranging from 0 to 10.

**Table 3:** Modified post-mortem graft maturation scoring system used in this study.

Score	Graft integrity	Synovial sealing at tunnel apertures	Graft vitality	Grafts in tunnels
0	Complete rupture (loss of continuity of all fibers)	<25% circumferential sealing	Non-vital graft	No graft in the tunnel
1	Partial rupture (loss of continuity of some fibers)	25%–75% circumferential sealing	Vital graft	Graft in one tunnel
2	No rupture (no loss of fiber continuity)	>75% circumferential sealing	-	Grafts present in two tunnels
3	-	-	-	Grafts present in all three tunnels

### Histopathological evaluation

Tissue samples were collected from the graft site, specifically from the region where the autologous lateral digital extensor muscle tendon graft was placed during surgery [18]. To ensure consistent sampling, tissue was harvested from the central portion of the grafted area, avoiding the edges where potential healing variations may occur. This site was chosen to assess the healing progression of the tendon graft itself and to evaluate the impact of PRP treatment. The samples were carefully excised and were immediately fixed in 10% buffered formalin solution for histopathological examination.

Following fixation, the samples were transferred to a 10% nitric acid solution for decalcification [19]. After adequate decalcification, tissue was returned to 10% buffered formalin for further fixation. The specimens were then dehydrated using increasing concentrations of alcohol (70%, 90%, and 100%). After dehydration, the samples were embedded in paraffin wax and sectioned serially in the cross-direction at 5.0 μm using a soft-tissue microtome (Leica Microsystems, Mussloch GmbH, Germany). The sections were stained with hematoxylin and eosin (H&E, Poly Scientific, Bayshore, NY).

The tissue sections were evaluated using a light microscope (Olympus U-MDOB, BX 40, Olympus Optical Co., Ltd., Japan) at various magnifications: 4×, 10×, 40×, and 100×. A board-certified pathologist assessed the presence and severity of hemorrhage, ossification, vascularization, and inflammation. Inflammation was characterized by polymorphonuclear neutrophils (PMNs), fibroblasts, osteoblasts, tenocytes, and bone-graft attachment. A histological grading system was developed for the evaluation of graft healing and attachment. This grading system consisted of eight parameters, each scored on a scale of 0 to 3, where a score of 0 indicates no changes, 1 indicates mild changes, 2 indicates moderate changes, and 3 indicates severe changes. The overall graft healing and attachment grade was determined by summing the scores for each parameter, with a total possible score of 24.

### Statistical analysis

Statistical analyses were conducted using a two-way analysis of variance to evaluate the effects of PRP treatment and time on various post-operative parameters. This method allowed the identification of differences between treatment groups and across time points, as well as any potential interactions between the two variables. Data were tested for normality using Q–Q plots before analysis, ensuring the validity of the results. The statistical approach aimed to discern the influence of PRP on post-operative recovery, graft healing, and histological outcomes while accounting for temporal changes in these parameters.

## RESULTS

### Lameness scores

In the control group (Group A), all animals had a grade 4 lameness score on day 10. By day 20, 75% remained at grade 4, whereas 25% improved to grade 3. On day 30, 75% of the patients showed grade 2 lameness, with the remaining 25% showed grade 3. By day 40, the distribution of scores varied: One-third were in Grade 1, another third in Grade 2, and the final third in Grade 3. In contrast, the PRP-treated group (Group B) had all animals at grade 4 lameness on days 10 and 20. By day 30, all animals had improved to grade 3. By day 40, 75% had grade 2 lameness, whereas 25% had grade 1.

### Post-operative complication scores

In this study, the most common post-operative complications were mild-to-moderate pain and swelling at the surgical site, with scores ranging from 1 to 2 on a scale of 0–3. Swelling at the surgical site did not exceed moderate levels and did not require additional treatment. Pain was managed effectively with routine post-operative analgesics. Mild crepitation was observed in some dogs, but none of the dogs developed infections.

Table 4 summarizes the effect of PRP treatment and time on post-operative complication scores in dogs that underwent CrCL repair using an autologous lateral digital extensor muscle tendon graft combined with PRP. A statistically significant difference (p = 0.0025) was observed between the control and PRP-treated groups, with the PRP-treated group showing fewer complications compared with the control group. Post-surgery time significantly influenced complication scores (p < 0.0001), indicating that recovery and complication development were time-dependent, with distinct differences at various time points. No statistically significant interaction between groups and subgroups was found (p = 0.2286), suggesting that the effect of PRP treatment was consistent across all time points.

**Table 4:** Effect of PRP treatment and time on post-operative complication scores in dogs that underwent cranial cruciate ligament repair using autologous lateral digital extensor muscle tendon graft.

Source	Degree of freedom	Sum of squares	F-ratio	Prob>F
Group	1	2.667	12.800	0.0025
Sub-group	3	94.333	150.933	<0.0001
Group versus subgroup	3	1.000	1.600	0.2286

PRP=Platelet-rich plasma

### Post-mortem graft maturation scores

Table 5 presents the effect of PRP treatment and time on post-mortem graft maturation scores in dogs that underwent CrCL repair using an autologous lateral digital extensor muscle tendon graft combined with PRP. No statistically significant difference (p = 1.0000) was found between the control and PRP-treated groups regarding post-mortem graft maturation, indicating that PRP treatment did not affect graft maturation.

**Table 5:** Effect of PRP treatment and time on post-mortem graft maturation scores in dogs that underwent cranial cruciate ligament repair using autologous lateral digital extensor muscle tendon graft.

Source	Degree of freedom	Sum of squares	F-ratio	Prob>F
Group	1	0.000	0.000	1.0000
Sub-group	3	23.333	8.485	0.0013
Group versus subgroup	3	1.333	0.485	0.6975

PRP=Platelet-rich plasma

However, the subgroups representing different time points significantly influenced graft maturation (p = 0.0013), indicating that graft maturation varied across the time points. The interaction between the groups and subgroups was not statistically significant (p = 0.6975), suggesting that the effect of PRP treatment on graft maturation was consistent across all time points and was not influenced by the timing of the evaluations.

### Histological evaluation

Table 6 shows the effect of PRP treatment and time on histological criteria scores in dogs that underwent CrCL repair using an autologous lateral digital extensor muscle tendon graft combined with PRP. A statistically significant difference (p = 0.0002) was observed between the control and PRP-treated groups, indicating that PRP treatment significantly improved histological outcomes. In addition, subgroups representing different time points showed a significant effect (p = 0.0029). However, the interaction between the groups and subgroups was not statistically significant (p = 0.3540), suggesting that the effect of PRP treatment on histological outcomes remained consistent across time without significant variation in the influence of time on the treatment effect.

**Table 6:** Effect of PRP treatment and time on histological criteria scores in dogs that underwent cranial cruciate ligament repair using autologous lateral digital extensor muscle tendon graft.

Source	Degree of freedom	Sum of squares	F-ratio	Prob>F
Group	1	73.500	22.329	0.0002
Sub-group	3	70.833	7.173	0.0029
Group vs. subgroup	3	11.500	1.165	0.3540

PRP=Platelet-rich plasma

**Figure 2:**
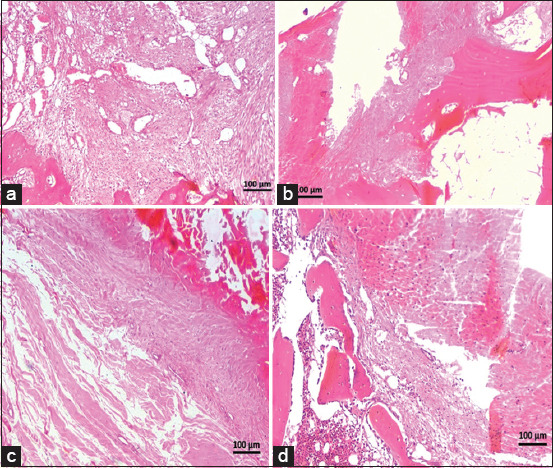
Micrographs of transverse sections of the femoral tunnel in the control group stained with hematoxylin and eosin at 10× magnification, showing the healing process over time. On day 10 (a), there was moderate hemorrhage with fibroblasts, osteoblasts, and tenocytes present at the graft-bone interface. By day 20 and day 30 (b and c), hemorrhage and ossification had decreased, with variable vascularization and cellular presence. On day 40 (d), both vascularization and graft-bone attachment were diminished.

**Figure 3:**
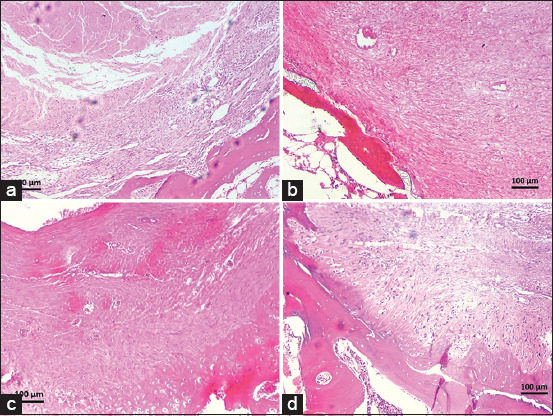
Micrographs of transverse sections of the 2^nd^ tibial tunnel in the platelet-rich plasma (PRP)-treated group stained with hematoxylin and eosin at 10× magnification, demonstrating the healing process at various time points. On day 10 (a), the PRP group exhibited more favorable early healing, with reduced hemorrhage and strong graft-bone attachment, along with a robust presence of tenocyte and osteoblasts. By day 20 (b), ossification and cellular activity had improved, with prominent osteoblasts and tenocytes. This trend of significant cellular presence was observed on day 30 (c). However, by day 40 (d), vascularization and graft attachment had declined.

Table 7 presents the histological observations of graft healing in the control and PRP groups at various time points. In the control group, day 10 observations showed moderate hemorrhage and the presence of fibroblasts, osteoblasts, tenocytes, and osteoblasts at the graft-bone interface (Figure 2a). On days 20 and 30, hemorrhage and ossification decreased, with variable vascularization and cellular presence (Figures 2b and c). On day 40, vascularization and graft-bone attachment were diminished (Figure 2d). In contrast, the PRP-treated group exhibited more favorable early healing. On day 10, hemorrhage was reduced, and there was a strong graft-bone attachment with a significant presence of tenocytes and osteoblasts at the tibial tunnel (Figure 3a). By day 20, improved ossification and cellular activity were observed, with prominent osteoblasts and tenocytes (Figure 3b). This trend continued, with a significant cellular presence on day 30 (Figure 3c). However, vascularization and graft attachment declined by day 40 (Figure 3d).

**Table 7:** Histological observations of graft healing at various time points in the control and PRP-treated groups.

Group	Time points (days)	Histological observations
Control	10	Moderate hemorrhage; presence of fibroblasts, osteoblasts, tenocyte, and osteoblasts at the graft-bone interface
	20	Decreased hemorrhage and ossification; variable vascularization, and cellular presence
	30	Decreased hemorrhage and ossification; continued variable vascularization and cellular presence
	40	Diminished vascularization and graft-bone attachment; less cellular activity
PRP-Treated	10	Reduced hemorrhage; strong graft-bone attachment with robust tenocyte and osteoblast presence at the tibial tunnel
	20	Improved ossification and cellular activity; prominent osteoblasts and tenocyte
	30	Continued significant cellular presence; well-developed graft-bone interface
	40	Declined vascularization and graft attachment, similar to the control group

PRP=Platelet-rich plasma

## DISCUSSION

This study presents a novel surgical technique for the management of CrCL rupture in dogs, which combines an autologous lateral digital extensor muscle tendon graft with PRP. The results showed that PRP treatment significantly enhanced recovery, as evidenced by improved lameness scores, histological outcomes, and fewer post-operative complications compared with the control group. Traditional surgical interventions for CrCL injuries, such as TPLO and TTA, face challenges, including high costs, invasiveness, and variable success rates [20–24]. These established techniques provide stable joint support but involve significant bone cutting, which increases the risk of complications and prolongs recovery time. In contrast, the novel technique described in this study requires minimal bone modification and the use of an autologous graft combined with PRP to promote healing. This approach could provide a more biologically favorable healing environment, potentially reducing the duration of recovery and improving long-term outcomes.

The PRP-treated group showed a more rapid improvement in lameness scores compared with the control group. While both groups started with a grade 4 lameness score, the PRP-treated group showed a faster recovery, with all animals improving to grade 3 by day 30 and the majority reaching grade 2 or grade 1 by day 40. In contrast, the control group exhibited a more gradual improvement, with a significant portion remaining at grade 3 and only a third of animals achieving grade 1 or 2 by day 40. These results are consistent with a previous study by Sharun *et al*. [25], which showed that PRP accelerates bone healing and manages non-union fractures. The benefits of PRP in managing osteoarthritis and enhancing graft maturation further support its effectiveness in improving lameness. A previous study by Conant *et al*. [26] has demonstrated that PRP injections for the treatment of partial ulnar collateral ligament tears in athletes led to higher return-to-competition rates and shorter recovery times compared with rehabilitation alone. Similar benefits in terms of reducing recovery times and improving outcomes suggest that PRP can also play a key role in enhancing graft integration and joint stability in CrCL injuries [26].

In this study, PRP treatment resulted in significantly lower post-operative complication scores compared with the control group. This finding is consistent with the established use of PRP as post-surgical supportive therapy, such as after femoral head osteotomy and TPLO, which was found to minimize post-operative complications and enhance recovery [25].

Histologically, results from this study revealed significant improvements in various histological parameters, such as hemorrhage reduction, decreased polymorphonuclear leukocytes (PMNs), increased osteoblasts, and enhanced bone attachment in the PRP-treated group. These results suggest the role of PRP in modulating inflammation and promoting tissue regeneration, which are critical for joint repair. These findings agree with a recent review article that highlighted PRP’s multifaceted role in wound healing through its composition, activation mechanisms, and clinical applications [27]. The aforementioned review clearly demonstrated PRP’s capacity to enhance healing in chronic and acute conditions, reduce complications, and support tissue regeneration [27]. This alignment with the broader literature suggests that PRP could be an essential therapeutic tool for improving patient outcomes and reducing healthcare costs.

The reduced number of PMNs in the PRP-treated group suggests effective inflammation control, whereas increased osteoblast activity indicates enhanced bone formation and graft integration. In addition, improved bone attachment results in a stronger and more secure graft connection, which is essential for long-term joint stability. These histological results align with the clinical benefits of PRP, as demonstrated by Le *et al*. [28], where PRP has been shown to improve outcomes in musculoskeletal conditions such as lateral epicondylitis and osteoarthritis of the knee, promoting better joint function, reduced lameness, and faster recovery.

Graft healing in ACL reconstruction has been conventionally categorized into three stages: Early healing, proliferation, and maturation [29]. The early healing phase is characterized by graft necrosis and hypocellularity, with no significant detectable revascularization occurring [29]. This is followed by the proliferation phase, marked by intense cell infiltration, and the maturation phase, which involves slow matrix remodeling. In ACL reconstruction using free tendon grafts, complete tunnel closure and ossification of the graft within the bone tunnels have not been fully achieved; rather, only partial incorporation of the graft into the tunnel wall has been observed [29]. In addition, the biological changes in the intra-articular region of the graft, which are often described as “ligamentization,” have not been fully realized [29]. Consequently, various biological modulation strategies have been proposed to enhance graft healing and improve clinical outcomes. A successful ACL repair with a tendon graft necessitates robust healing within the bone tunnel and complete “ligamentization” in the intra-articular region as soon as possible post-surgery, as this is crucial for facilitating early and aggressive rehabilitation and a rapid return to full activity.

In this study, gross evaluation of the surgical site revealed no significant difference in post-mortem graft maturation scores between the PRP-treated and control groups (p = 1.0000). This finding suggests that although PRP treatment may enhance other aspects of recovery, it did not have a measurable effect on graft maturation. However, the significant variation observed across different time points indicates that graft maturation is inherently time-dependent, emphasizing the need for continued focus on optimizing biological interventions to facilitate timely graft integration and functional recovery in CrCL reconstruction.

The practical implications of this novel surgical technique are significant for veterinary practice, offering a less invasive alternative to traditional methods such as TPLO and TTA. In comparison, this technique can reduce the recovery time, improve joint stability, and lower the risk of complications. The procedure was well tolerated, with no significant intraoperative complications. Although the surgical duration was slightly longer due to additional graft preparation and PRP application, the enhanced healing observed in the PRP-treated group may offset this increased time. This approach creates a more biologically favorable healing environment, which improves outcomes.

## CONCLUSION

This study introduces a novel surgical approach for CrCL repair in dogs, combining an autologous lateral digital extensor muscle tendon graft with PRP. The findings revealed significant enhancements in tissue healing with PRP, demonstrated by improved histological scores and reduced post-operative complications. Although graft maturation was unaffected by PRP, time played a critical role in the healing process. The technique provides a less invasive, cost-effective alternative to traditional CrCL repair methods such as TPLO and TTA, with the added benefit of PRP’s biological healing properties.

The study’s strengths lie in its innovative approach, robust statistical analysis, and comprehensive evaluation of clinical, histological, and post-mortem parameters. However, limitations include a small sample size and reliance on surgically induced CrCL rupture, which may not fully represent naturally occurring conditions. In addition, the short-term follow-up restricts insights into long-term outcomes.

Future research should explore the technique’s efficacy in a broader and more diverse population, assess long-term outcomes such as joint stability and osteoarthritis, and refine PRP application methods to maximize its therapeutic potential. This study highlights the promise of integrating autologous grafting with PRP, contributing to advancements in veterinary orthopedic surgery and improving treatment options for CrCL injuries in dogs.

## AUTHORS’ CONTRIBUTIONS

MHD, ZBI, and MAA: Conceived and designed the study. MAM: Project management and revision. ZBI, MAM, and MAA: Critically reviewed and modified the manuscript. ZBI and MAM: Performed final manuscript revision. HMH: Performed statistical analysis and data interpretation and prepared the tables and figures. SR: Conducted comprehensive literature search, performed the study, collected data, and drafted the manuscript. All authors have read and approved the final manuscript.
